# Circulating noncoding RNAs as early predictive biomarkers in preeclampsia: a diagnostic meta-analysis

**DOI:** 10.1186/s12958-021-00852-8

**Published:** 2021-12-01

**Authors:** Sha Su, Fang Yang, Linlin Zhong, Lihong Pang

**Affiliations:** Department of Obstetrics and Gynecology, The First Affiliated Hospital of Guangxi Medical University, Shuangyong Road, Nanning, 530021 Guangxi China

**Keywords:** ncRNAs, Preeclampsia, Biomarkers, Diagnostic meta-analysis

## Abstract

**Background:**

We designed a meta-analysis to evaluate the clinical significance and efficacy of circulating noncoding RNAs (ncRNAs) in the early prediction of preeclampsia.

**Methods:**

PubMed, Embase and the Cochrane Library were used to search for literature. The combined prediction performance was evaluated by calculating the area under the summary receiver operator characteristic (SROC) curve. The potential sources of heterogeneity were analysed by meta-regression analysis and subgroup analysis. All statistical analyses and mapping were performed by RevMan 5.3 and Stata 12.0.

**Results:**

A total of 41 studies from 14 articles, including 557 preeclampsia patients and 842 controls, were included in our meta-analysis. All studies collected blood before onset. NcRNAs in blood performed relatively well in predicting preeclampsia. The combined sensitivity was 0.71, the specificity was 0.84, and the area under the SROC curve (AUC) was 0.86. Peripheral blood mononuclear cell (PBMC) samples showed the best diagnostic accuracy. The combined AUC was 0.93. Combined detection was better than single detection, and miRNA was better than circRNA. The heterogeneity of the study was determined by sample size, lncRNA characteristics, lncRNA source and race.

**Conclusion:**

Circulating ncRNAs can be valuable biomarkers used as candidates for noninvasive early predictive biomarkers of preeclampsia and have great clinical application prospects. The clinical value of ncRNAs needs to be tested by further multicentre, comprehensive and prospective studies, and the test criteria should be established.

**Supplementary Information:**

The online version contains supplementary material available at 10.1186/s12958-021-00852-8.

## Background

Preeclampsia remains a main cause of maternal and perinatal mortality and morbidity, with a global incidence rate of 2–8%. It is a pregnancy-specific disease characterized by new onset hypertension and proteinuria, sometimes progressing into multiple organ damage [1, 2]. How to effectively predict preeclampsia in the early stage of disease is an important topic, because necessary measures can be taken as soon as possible to prevent or at least reduce the frequency and severity of PE [3]. To date, the pathogenesis of preeclampsia has not been fully elucidated, and epigenetic changes play a crucial role in the development and progression of PE disease, including noncoding RNA (ncRNA) regulation [4, 5]. ncRNAs can be classified into several types depending on their length or structure, such as long noncoding RNAs (lncRNAs), microRNAs (miRNAs) and circular RNAs (circRNAs). These types of RNA are present in circulation and tissue, intracellular and extracellular, and thus, they have been used as biomarkers of different diseases [6]. Numerous recent studies have demonstrated the applicability of circulating ncRNA in PE [7, 8], although the conclusions are inconsistent and the mechanism is unclear. Can circulating ncRNA be a potential biomarker for the early prediction of preeclampsia? Several relevant meta-analyses and reviews evaluated the relationship between miRNA, circRNA and lncRNA preeclampsia separately [9–12]. In our study, we utilized the method for systematic reviews of diagnostic test accuracy to quantitatively evaluate the diagnostic value of different ncRNAs as circulating biomarkers for PE.

## Methods

### Databases search

We searched literature databases (PubMed, Embase and Cochrane Library) to identify relevant studies published through June 1, 2021. We used the following search terms to retrieve relevant data: (“pre-eclampsia“ OR “pre eclampsia” OR “preeclampsia” OR “eclampsia” OR “gestational hypertensive disorder” OR “pregnancy hypertension” OR “hypertensive disorders of pregnancy” OR “pregnancy-induced hypertension” OR “pregnancy-associated hypertension”) AND (“microRNAs” OR “microRNA” OR “miRNAs” OR “miRNA” OR “miR”) AND (“ROC” OR “sensitivity” OR “specificity”); (“pre-eclampsia” OR “pre eclampsia” OR “preeclampsia” OR “eclampsia” OR “gestational hypertensive disorder” OR “pregnancy hypertension” OR “hypertensive disorders of pregnancy” OR “pregnancy-induced hypertension” OR “pregnancy-associated hypertension”) AND (“circular RNA” OR “circRNA” OR “circRNAs“) AND (“ROC” OR “sensitivity” OR “specificity”); (“pre-eclampsia” OR “pre eclampsia” OR “preeclampsia” OR “eclampsia” OR “gestational hypertensive disorder” OR “pregnancy hypertension” OR “hypertensive disorders of pregnancy” OR “pregnancy-induced hypertension” OR “pregnancy-associated hypertension”) AND (lncRNAs” OR “RNA, long noncoding” OR “long noncoding RNA” OR “lncRNA”) AND (“ROC” OR “sensitivity” OR “specificity”);

### Eligibility criteria

Studies were considered eligible if they met the following criteria: 1) predictive capacity of miRNA、circRNA、lncRNA for PE was provided; 2) the acknowledged gold reference standard was used to make diagnosis of PE patients: blood pressure 140/90 mmHg and proteinuria 0.3 g/day after 20 gestational weeks; 3) all women had singleton pregnancies, and those with pregnancy complications were excluded; 4) pregnant women with no signs or symptoms of preeclampsia at the time of sampling; and 5) FP, TP, FN and TN were provided to construct the 2 × 2 contingency table. The exclusion criteria were as follows: 1) written in a language other than English; 2) the expression level of ncRNA was obtained from cell lines or animals; 3) reviews, letters, and meeting records; 4) studies focusing on gene polymorphisms; and 5) studies with insufficient data.

### Data extraction and quality assessment

Articles were independently screened by two researchers, and disagreements were resolved by consulting a third researcher. The extracted data from these articles included first author, year of publication, country, sample size, time of sampling, type of PE, sample type, internal reference gene, ncRNA profiling, diagnostic value (sensitivity, specificity and AUC) and expression level. The two aforementioned researchers used the QUADAS-2 score system [13] to assess the quality of the included articles, and a third researcher resolved the discrepancies.

### Statistical analysis

We extracted the TP, FP, FN, and TN of each study to calculate the pooled sensitivity, specificity, PLR, NLR, DOR, and corresponding 95% CI. We also tested the pooled diagnostic value of ncRNAs by examining the SROC curve and the area under the SROC curve (AUC). In the present study, Deeks’ funnel plot was also conducted to test publication bias. We assessed heterogeneity among the studies using the chi-squared test and I^2^ statistic. If *P* < .1 or I^2^ > 50%, heterogeneity was defined as significant. We also conducted meta-regression, subgroup and sensitivity analyses to identify potential sources of heterogeneity. We carried out all analyses using Review Manager 5.3 (The Nordic Cochrane Centre, The Cochrane Collaboration, London, UK) and Stata 12.0 (StataCorp, College Station, TX, USA), and a value of *P* < .05 was considered statistically significant.

## Results

### Literature search

**Fig. 1:**
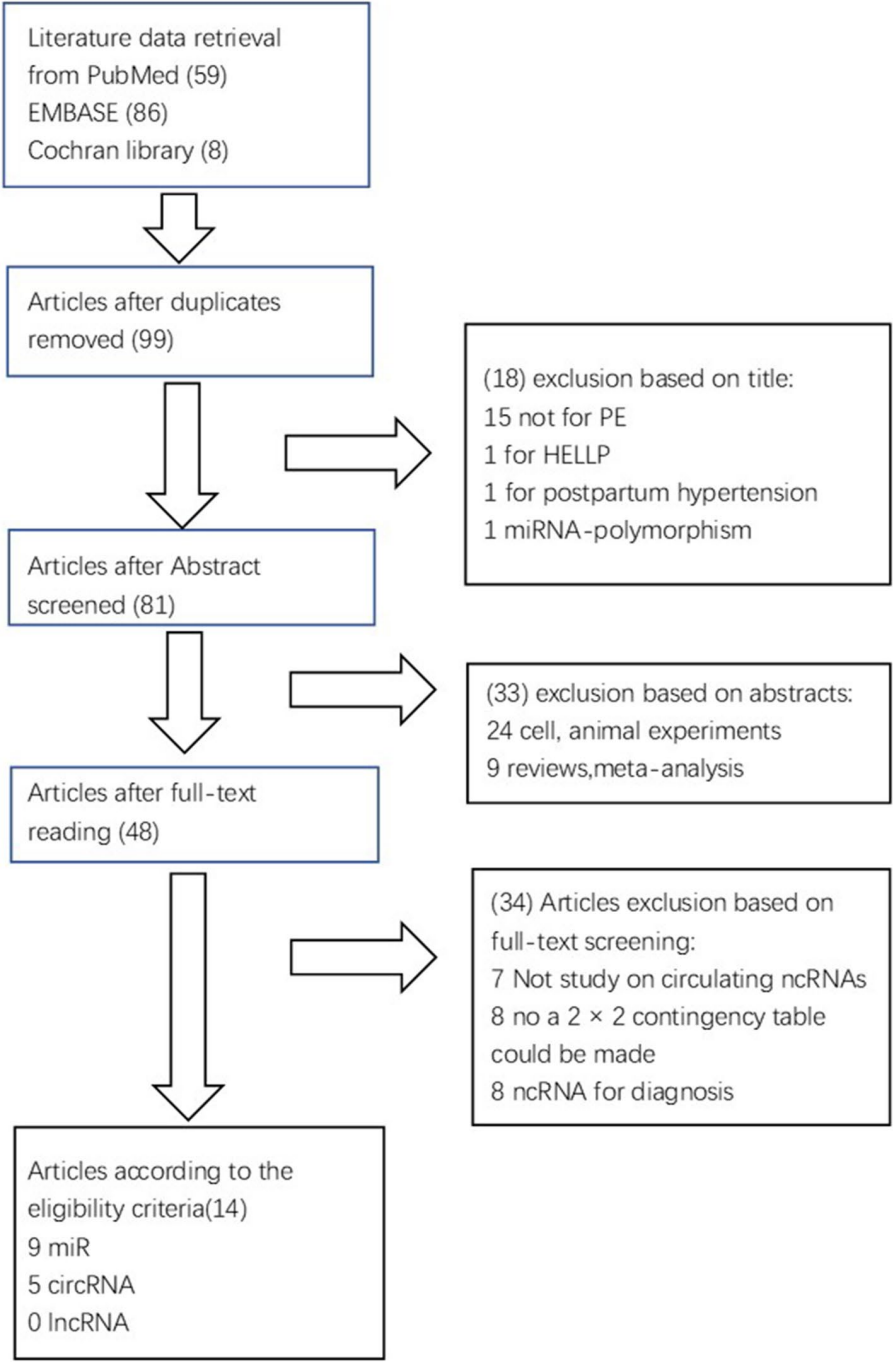
Flow chart for selection of eligible articles

We searched 153 records in PubMed, Embase, and the Cochrane Library. Of these, 54 duplicate studies were excluded. We excluded 18 records after reading the titles and 33 records after reviewing the abstracts. Subsequently, we assessed the full texts of the remaining 48 articles and excluded 34 studies based on the exclusion criteria, including 7 not studies on circulating ncRNAs, 8 without clinical data to make a 2 × 2 contingency table and 8 studies that used ncRNAs for diagnosis. In total, 14 studies were ultimately included in this study [14–27]. The selection process flow chart of our study is presented in Fig.1.

### Literature characteristics and quality assessment

Additional file 1(Table 1) demonstrates the general characteristics of the included articles. A total of 41 studies from 14 articles published from 2012 to 2021 were included in our meta-analysis, including 842 controls and 557 PE patients. qRT–PCR was utilized to detect the expression level of ncRNAs in all included studies. Sources of ncRNAs included plasma, serum, plasma exosomes and PBMCs. Most studies of blood samples were collected before 20 weeks of gestation. Ten studies evaluated the predictive value of circulating circRNAs for preeclampsia, while 31 studies evaluated circulating miRNAs for preeclampsia. Thirteen studies used combined marker assays, including multiple ncRNAs. Twenty-eight studies used a single marker assay. Seven articles differentiated the type of PE, including EOPE and LOPE, as mild and severe. Fifteen studies from China involved Chinese participants. The majority of studies adopted in this meta-analysis met at least four criteria outlined in the QUADAS-2 tool. A main limitation of this study is that most studies adopted a case-control design, and blinding was not used in the evaluation of test results. However, in general, the overall quality of the studies was acceptable.

### Predictive efficacy of circulating ncRNAs for PE

**Fig. 2:**
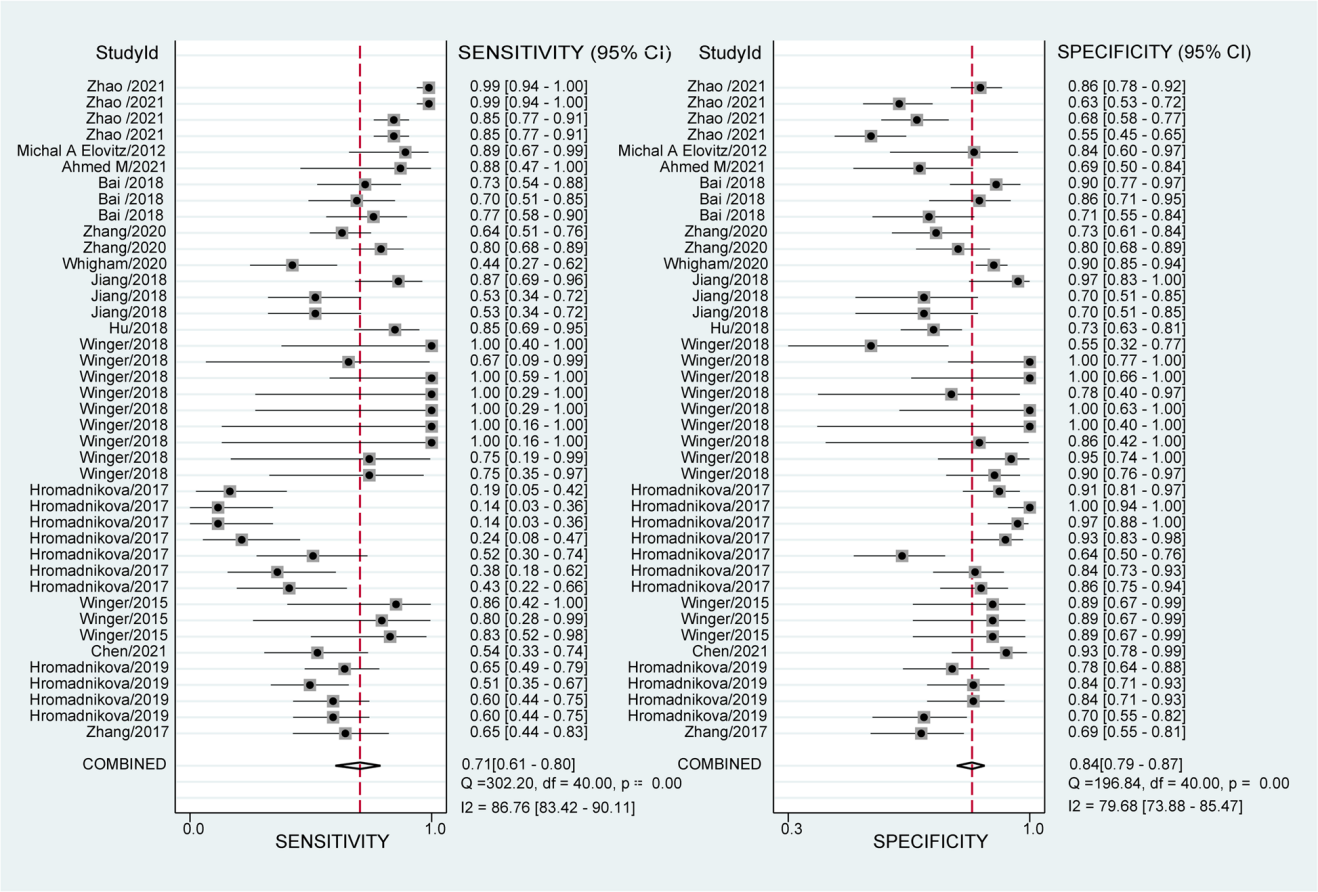
Forest plots of the pooled sensitivity and specificity of overall studies

**Fig. 3:**
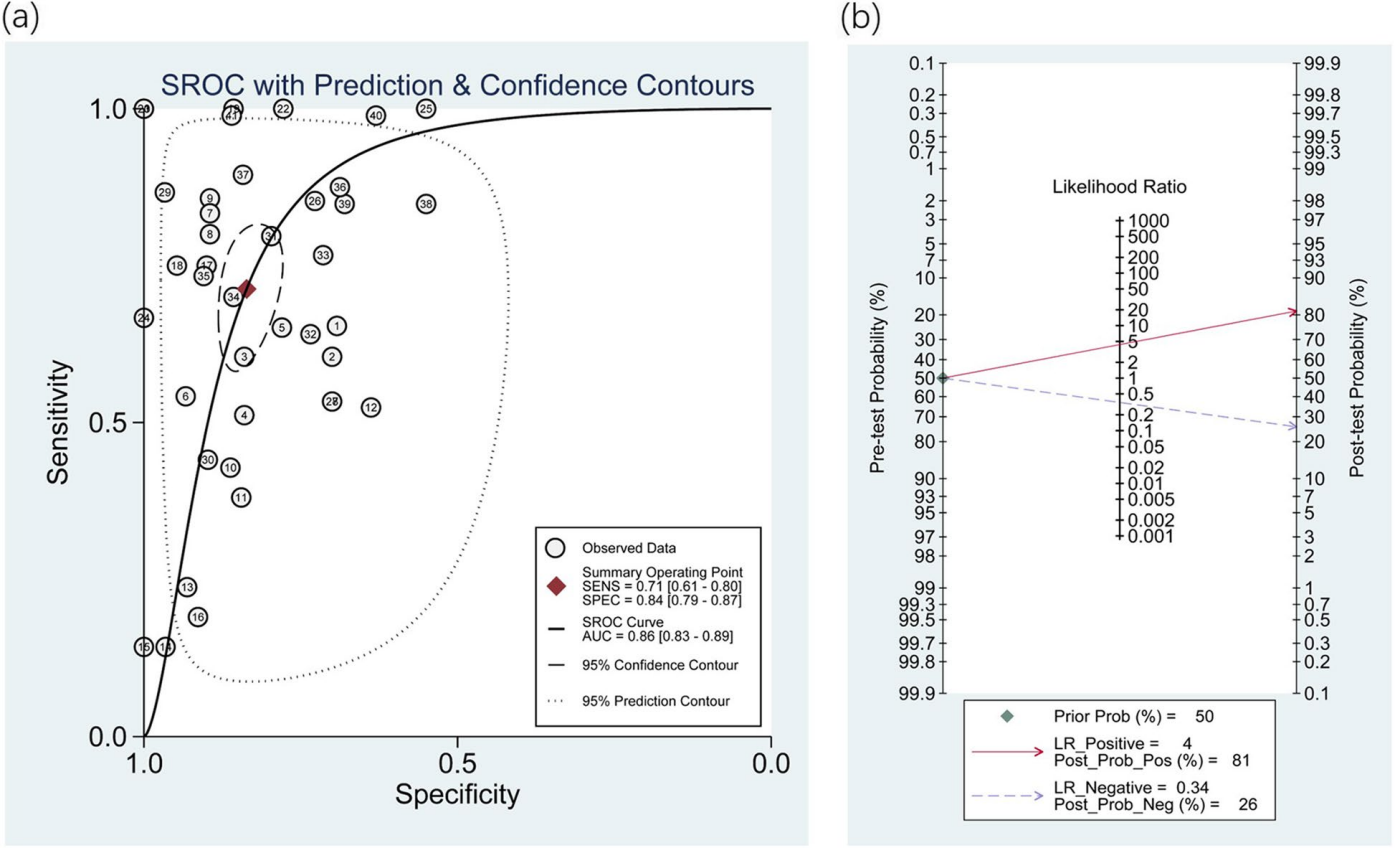
Predictive performance of circulating lncRNAs of overall studies for PE. (**a**) SROC curve. (**b**) Fagan’s nomogram

Since there was significant heterogeneity among studies in sensitivity (I2 = 86.76%) and specificity (I2 = 79.68%) (*P* < 0.01)(Fig.2),a random effects model was used. As presented in Additional file 2(Table 2), the pooled parameters determined from all 41 studies were sensitivity, 0.71 (95% CI 0.61, 0.80); specificity, 0.84 (95% CI 0.79, 0.87); PLR, 4.4 (95% CI 3.5, 5.5); NLR, 0.34 (95% CI 0.25, 0.47); DOR, 13 (95% CI 8, 20); and AUC, 0.86 (95% CI 0.83, 0.89) (Fig.3(a)), signifying that ncRNAs in circulation may serve as a good predictive index for PE with high accuracy. As demonstrated in Fagan’s plot (Fig.3 (b)), the pretest probability was 50%, the posttest probability of PE for a positive test result was 81%, and the negative test result was 26%, indicating that both the posttest probabilities and likelihood ratios were high. The PLR of 4.4 showed that a person with PE is 4.4 times more likely to have a positive test result than a healthy person. Furthermore, the DOR value was 13 (95% CI8, 20), which revealed that ncRNAs in circulation can be used to distinguish PE patients from controls.

### Subgroup analysis

**Fig. 4:**
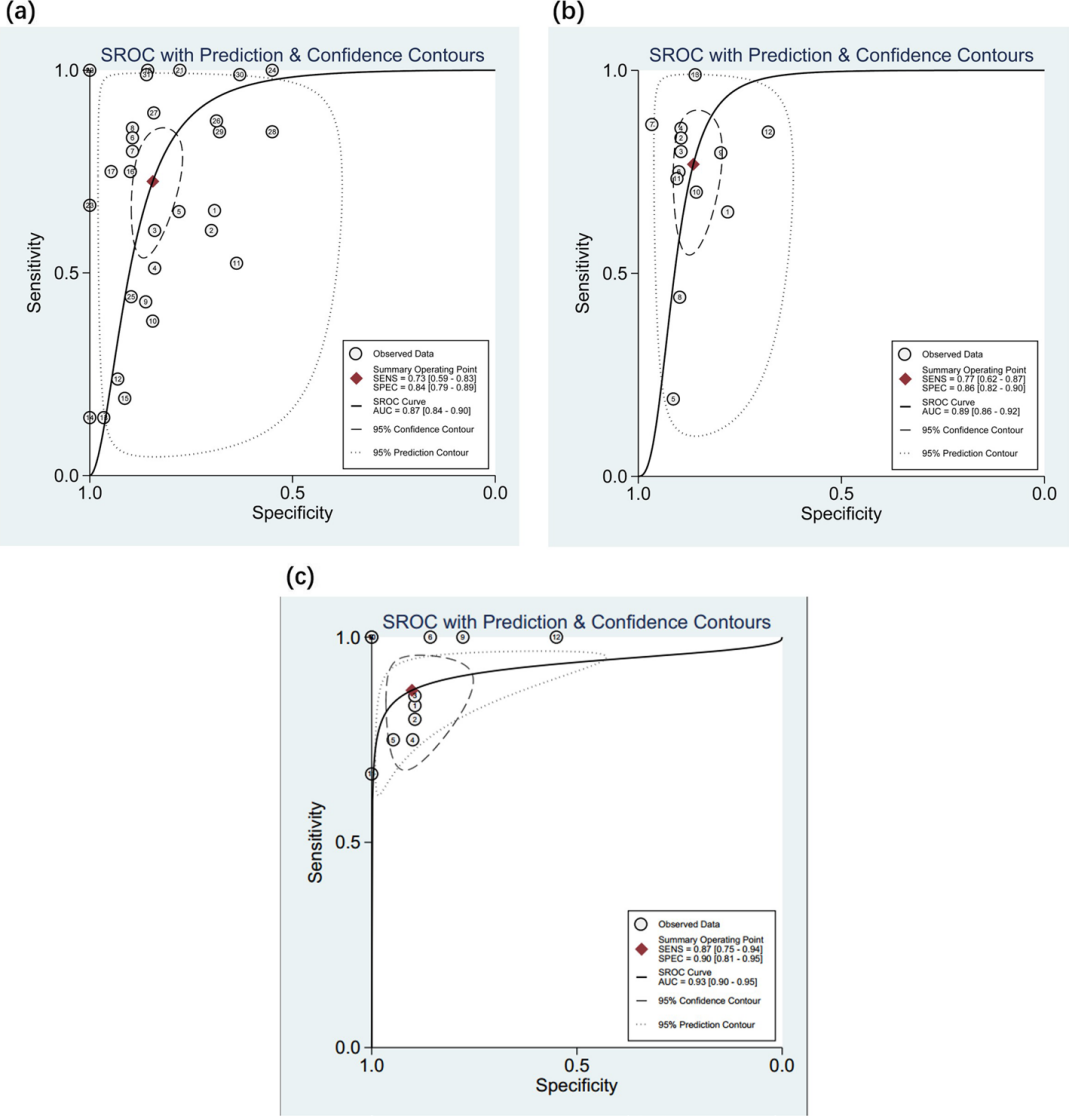
SROC curves based on predictive studies of (**a**) miRNA, (**b**) combined ncRNA assay, and (**c**) PBMCs

There are multiple potential sources of heterogeneity, so we performed subgroup analysis. The pooled results for diagnostic value in different subgroups are shown in Additional file 2(Table 2). circRNAs yielded a pooled AUC of 0.83, while miRNA yielded a pooled AUC of 0.87. The combined ncRNA assay exhibited good diagnostic accuracy with a pooled AUC of 0.89, while the single ncRNA assay had a pooled AUC of 0.82. PBMCs had an AUC of 0.93(Fig.4), and plasma had an AUC of 0.80. In addition, Chinese-based and EOPE ncRNA assays yielded a pooled AUC of 0.84. Generally, each subgroup analysis had a good predictive effect.

### Regression analysis and sensitivity testing

**Fig. 5:**
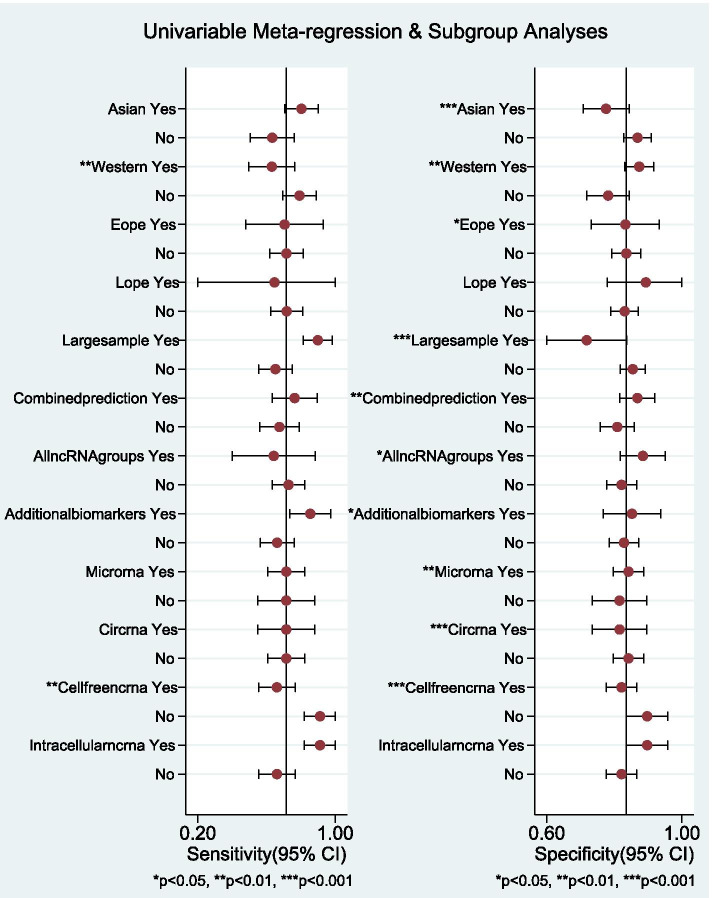
Regression analysis and subgroup analysis

**Fig. 6:**
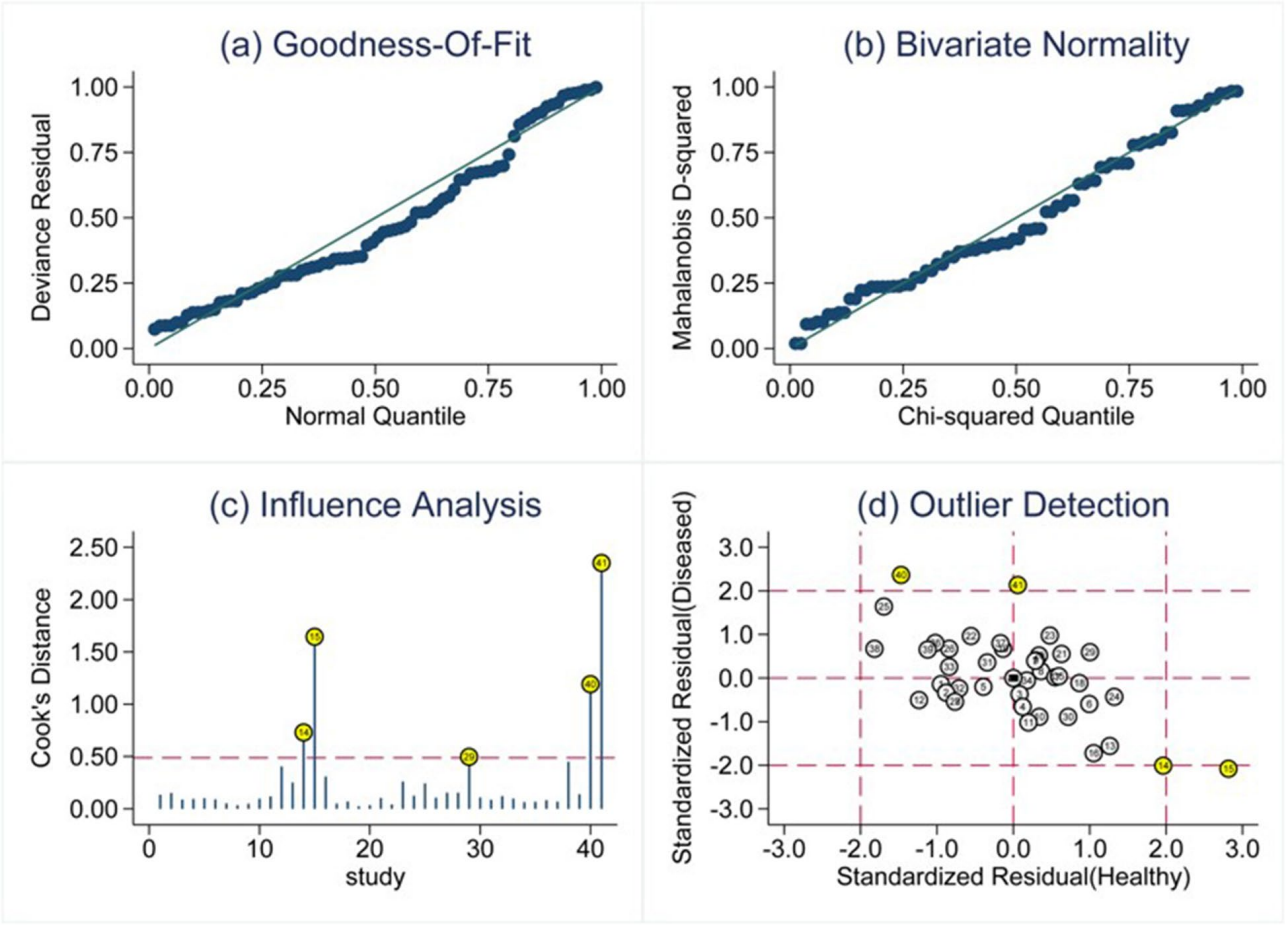
Impact analysis and outlier detection. (**a**) Goodness of fit (**b**) bivariate normality (**c**) impact analysis, and (**d**) outlier detection

**Fig. 7:**
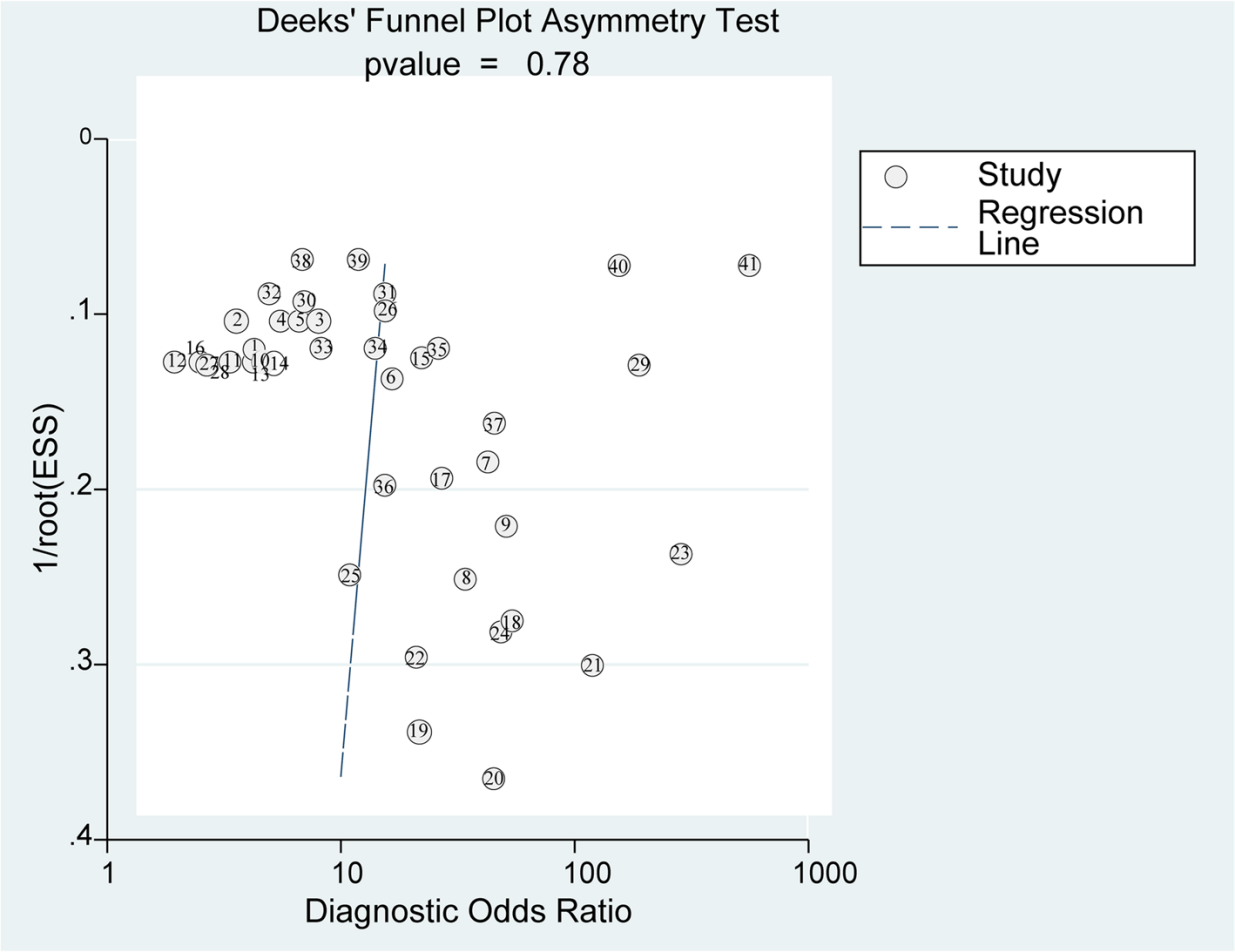
Deeks’ funnel plot symmetry test for publication bias

The potential sources of heterogeneity were further explored through meta-regression analysis (Fig.5). As displayed in Fig, sample size, ncRNA species, lncRNA profiling (single or combined, All-ncRNA or additional biomarkers), specimen types and ethnicity seemed to be the primary sources of heterogeneity for ncRNA assays in PE. We performed sensitivity analysis to understand the combined effect size changes after removing an individual study (Fig.6). Our results showed that the bivariate model was moderately robust. Five outliers were identifed by impact analysis. Four outliers were found through outlier detection. Deeks’ funnel plot symmetry test (Fig.7) was used to assess potential publication bias. In this study, the *P* value of linear regression was 0.78, which indicated no publication bias.

## Discussion

The prediction of preeclampsia is of great significance for early prevention and treatment and reducing maternal and infant mortality. In current clinical guidelines,all pregnant women should be screened for PE in the first trimester of pregnancy. For women who are at high risk of developing PE, attention should be given to the early signs of PE throughout pregnancy [3].. It is well known that maternal high-risk factors are a common means of screening PE, including advanced maternal age; nulliparity; previous history of PE; short and long interpregnancy interval; use of assisted reproducing technologies; family history of PE; obesity; and comorbid medical conditions. However, maternal high-risk factors are unlikely to be effective in predicting the onset of PE. Researchers have suggested that [28, 29] combinations of tests such as the mean arterial pressure (MAP) measurement, the soluble Fms-like tyrosine kinase-1/placental growth factor ratio (sFlt-1/PlGF) and uterine artery pulsatility index (UTPI) raise the effectiveness of screening. With the development of epigenetics and molecular biology, increasing evidence has shown that noncoding RNAs (ncRNAs) guide and regulate a large number of biological processes. Many ncRNAs, including microRNAs and long noncoding transcripts, show almost complete or major expression in the placenta and show altered expression patterns in the placenta during complex pregnancy [4, 5, 30, 31]. Our study evaluated the ability of circulating noncoding RNA to predict preeclampsia and explored its possibility as a noninvasive biomarker of PE.

Previously, Yin [32] studied the ability of circulating miRNAs as biomarkers for the prediction of preeclampsia. Three articles were included in the meta-analysis, with publication bias. The AUC of miRNAs for the prediction of PE was 0.69. In our studies, we included 14 articles, including cyclic RNA and miRNAs, which was more comprehensive. Our report verified the potential predictive performance of ncRNAs in blood as noninvasive biomarkers with a pooled AUC value of 0.86 (pooled sensitivity = 71%; pooled specificity = 84%). Moreover, the DOR value of 13 (95% CI 11, 19) signified that circulating ncRNA test-positive patients have a 13-fold higher chance of developing PE than controls.

Ten studies examined the predictive value of circulating circRNAs in PE, while 31 studies examined circulating miRNAs. miRNAs were stronger predictors, with a pooled AUC of 0.87, a sensitivity of 0.73, a specificity of 0.84, a PLR of 4.7, an NLR of 0.32, and a DOR of 14, thereby exhibiting relatively high predictive accuracy. In our study, we did not retrieve relevant studies on the early prediction of preeclampsia by circulating lncRNAs. Thirteen studies involved a combined ncRNA assay, and 28 studies referred to a single ncRNA assay; the combined ncRNA assay performed better, with a pooled AUC of 0.89, a sensitivity of 0.77, a specificity of 0.86, a PLR of 5.7, an NLR of 0.27, and a DOR of 21. The combined strategy includes not only multiple ncRNAs but also well-known biomarkers, such as sFlt-1/PlGF and pregnancy associated plasma protein A (PAPP-A). Finally, subgroup analysis based on these parameters is limited due to insufficient published data.

Then, subgroup analyses and meta-regression analysis were carried out to explore the potential sources of the heterogeneity. Our results demonstrated that both pooled sensitivity and specificity were influenced by sample size, ncRNA species, lncRNA profiling (single or combined), specimen type and ethnicity, indicating that the factors discussed above may be the principal sources of heterogeneity for ncRNA assays in PE.

We performed the best possible analyses; however, our report is not perfect, mainly due to significant statistical heterogeneity. The sources of heterogeneity include sample size, lncRNA characteristics, lncRNA source and race, which are inevitable, and thus, the results were affected. In addition, the sensitivity and specificity of this test make it difficult to say that it is useful as a predictive marker for preeclampsia. It should be combined with other markers such as maternal risk factors,uterine artery Doppler measurement results and arterial pressure, to show its superiority over more convenient tests. Furthermore,there are individual differences in the clinical manifestations and organ involvement of preeclampsia. The differences in the expression of noncoding RNA in different gestational cycles and the interference of various internal and external factors during pregnancy may affect the accuracy of the data. The effectiveness of circulating noncoding RNA in the early prediction of preeclampsia is promising. We need larger multicentre sample studies to verify this hypothesis. Additionally, it is wise to identify recognized endogenous reference genes, detection reagents and methods and establish standardized ncRNAs.

## Conclusion

Our meta-analysis results showed that circulating ncRNAs can be valuable biomarkers used as candidates for noninvasive early predictive biomarkers of preeclampsia and have great clinical application prospects. The clinical value of ncRNAs needs to be tested by further multicentre, comprehensive and prospective studies, and the test criteria should be established.

## Supplementary Information

**Additional file 1.** 

**Additional file 2.** 

## Data Availability

The original data from the survey is available.

## Abbreviations

PE: Preeclampsia
EOPE: Early onset preeclampsia
LOPE: Late onset preeclampsia;
ncRNA: Non-coding RNA
miRNA: microRNA
lncRNA: Long non-codingRNA
PBMC: Peripheral blood mononuclear cell
TP: True positive
FP: False positive
FN: False negative
TN: True negative
SEN: Sensitivity
SPE: Specificity
AUC: Area under the curve
SROC: Summary receiver operator characteristic
NA: Not available
